# Differential Methylation in the *GSTT1* Regulatory Region in Sudden Unexplained Death and Sudden Unexpected Death in Epilepsy

**DOI:** 10.3390/ijms22062790

**Published:** 2021-03-10

**Authors:** Steffan Noe Christiansen, Stine Bøttcher Jacobsen, Jeppe Dyrberg Andersen, Marie-Louise Kampmann, Linea Christine Trudsø, Kristine Boisen Olsen, Jacob Tfelt-Hansen, Jytte Banner, Niels Morling

**Affiliations:** 1 Section of Forensic Genetics, Department of Forensic Medicine, Faculty of Health and Medical Sciences, University of Copenhagen, DK-1353 Copenhagen, Denmark; steffan.christiansen@sund.ku.dk (S.N.C.); jeppe.dyrberg.andersen@sund.ku.dk (J.D.A.); marie.louise.kampmann@sund.ku.dk (M.-L.K.); niels.morling@sund.ku.dk (N.M.); 2 Department of Biomedical Sciences, Faculty of Health and Medical Sciences, University of Copenhagen, DK-1353 Copenhagen, Denmark; linea.trudsoe@sund.ku.dk; 3 Section of Forensic Pathology, Department of Forensic Medicine, Faculty of Health and Medical Sciences, University of Copenhagen, DK-1353 Copenhagen, Denmark; kbolsen@sund.ku.dk (K.B.O.); jacob.tfelt@regionh.dk (J.T.-H.); jytte.banner@sund.ku.dk (J.B.); 4 The Heart Centre, Department of Cardiology, Copenhagen University Hospital Rigshospitalet, DK-1353 Copenhagen, Denmark

**Keywords:** epigenetics, genetics, DNA methylation, RNA, molecular autopsy, sudden cardiac death, sudden unexplained death, sudden unexpected death in epilepsy

## Abstract

Sudden cardiac death (SCD) is a diagnostic challenge in forensic medicine. In a relatively large proportion of the SCDs, the deaths remain unexplained after autopsy. This challenge is likely caused by unknown disease mechanisms. Changes in DNA methylation have been associated with several heart diseases, but the role of DNA methylation in SCD is unknown. In this study, we investigated DNA methylation in two SCD subtypes, sudden unexplained death (SUD) and sudden unexpected death in epilepsy (SUDEP). We assessed DNA methylation of more than 850,000 positions in cardiac tissue from nine SUD and 14 SUDEP cases using the Illumina Infinium MethylationEPIC BeadChip. In total, six differently methylated regions (DMRs) between the SUD and SUDEP cases were identified. The DMRs were located in proximity to or overlapping genes encoding proteins that are a part of the glutathione S-transferase (GST) superfamily. Whole genome sequencing (WGS) showed that the DNA methylation alterations were not caused by genetic changes, while whole transcriptome sequencing (WTS) showed that DNA methylation was associated with expression levels of the *GSTT1* gene. In conclusion, our results indicate that cardiac DNA methylation is similar in SUD and SUDEP, but with regional differential methylation in proximity to GST genes.

## 1. Introduction

The sudden and unexpected death of a young individual is a devastating event in families and remains a key challenge to healthcare systems and forensic examinations worldwide. In some cases, death is the first disease manifestation, and this demonstrates that timely diagnosis is essential to prevent premature death [1,2]. The majority of sudden cardiac deaths (SCDs) in the young are suspected to result from undiagnosed structural cardiovascular disorders that can be identified at post-mortem investigations [3,4]. However, in cases of a structurally normal heart, routine autopsies often fail to identify a disease as the cause of death. These sudden unexplained deaths (SUDs) are presumed to be caused by undiagnosed cardiac arrhythmias and are, in the case of a negative histological and toxicological investigation, termed sudden arrhythmic death syndrome [5,6]. Diseases most commonly associated with SUD are inherited cardiac channelopathies such as long QT syndrome (LQTS), Brugada Syndrome (BrS), and catecholaminergic polymorphic ventricular tachycardia (CPVT) [7,8]. Extensive research on the genetics of inherited cardiac diseases have been performed and several studies report successful identification of causal genetic variants in relation to SUD [9,10,11]. Furthermore, an association between methylation of DNA and cardiac disease has been suggested [12,13,14,15]. However, the role of epigenetic patterns in SUD remains largely undetermined.

Epilepsy, diabetes, and psychiatric diseases have also been associated with a higher risk of SCD in young individuals [16,17,18]. Sudden unexpected death in epilepsy (SUDEP) accounts for the majority of premature mortality among patients with epilepsy [19,20]. SUD and SUDEP share common circumstances of death with both events occurring predominantly during sleep or at rest [21,22,23]. This may indicate similar pathophysiological mechanisms. Despite extensive research, the etiology of SUDEP remains largely unknown. Several risk factors have been suggested with the most predominant being the presence of generalised tonic-clonic seizures [24,25]. However, a combination of neurological, cardiac, and respiratory factors has also been proposed to contribute to death [22,26]. Cardiac channelopathies may predispose to SUDEP as channelopathies can present with both arrhythmia and seizure [27,28,29].

A genetic basis of SUDEP has been suggested, and several studies have identified pathogenic variants in genes co-expressed in the brain and the heart [30,31,32,33,34]. However, the potential contribution of epigenetic factors to the risk of SUDEP as well as the effect of gene expression levels remain largely unknown.

Epigenetic patterns are tissue specific and, hence, studies of epigenetic phenomena in various tissues require access to the tissue of interest. A major limitation of epigenetic studies of heart diseases is, therefore, the general unavailability of cardiac tissue. However, in post-mortem investigations, the accessibility of tissues from autopsied individuals offers a unique opportunity for epigenetic investigations of cardiac tissue. A key challenge of post-mortem genetic and epigenetic testing is, though, the post-mortem degradation of the tissue. Post-mortem decomposition may result in widespread fragmentation of nucleic acids [35]. The quality of the nucleic acids can, furthermore, be reduced by the tissue storage methods. Formalin fixed and paraffin embedded (FFPE) tissue has for many years been widely used as preparation for histological examinations and long-term storage. Unfortunately, FFPE tissue may induce sequence artefacts [36,37].

In this study, we investigated DNA methylation in FFPE cardiac tissues from SUD and SUDEP cases and identified differentially methylated regions (DMRs) between the two groups. The heart tissue from SUDEP victims was also examined for RNA expression. The results were compared to DNA variation investigated using whole genome sequencing (WGS).

## 2. Results

### 2.1. Characteristics of the Study Population

The study included ten males and four females diagnosed with SUDEP and six males and three females diagnosed with SUD (Table 1). The median age was 35 years in both groups with the ranges 0–50 years and 3–49 years among the SUD and SUDEP cases, respectively. Deaths in both groups were predominantly unwitnessed and occurred during sleep or rest. There were no significant differences between the two investigated groups except for the difference in the expected detected antiepileptic drugs (Table 1).

### 2.2. Allele Frequencies in Sudden Unexplained Death and Sudden Unexpected Death in Epilepsy

The allele frequencies of variants in SUD and SUDEP cases were assessed. No statistically significant difference between SUD and SUDEP was identified.

### 2.3. Principal Component Analysis

**Figure 1:**
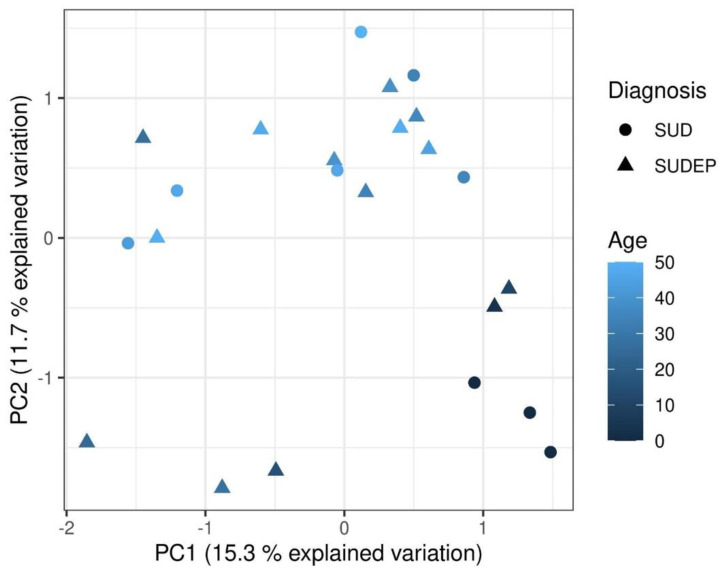
Principal component analysis plot. The principal component analysis was based on M-values. The samples are coloured according to age and the shape of the symbols shows diagnosis. PC: Principal component. SUD: sudden unexplained death. SUDEP: sudden unexpected death in epilepsy.

The principal component analysis (PCA) was conducted to investigate the main patterns in the DNA methylation data (Figure 1). The analysis revealed that PC2 tended to separate older individuals from younger individuals. However, the analysis revealed no overall difference between SUDEP and SUD.

### 2.4. Regulatory Association of the GSTT1 DMR

**Figure 2:**
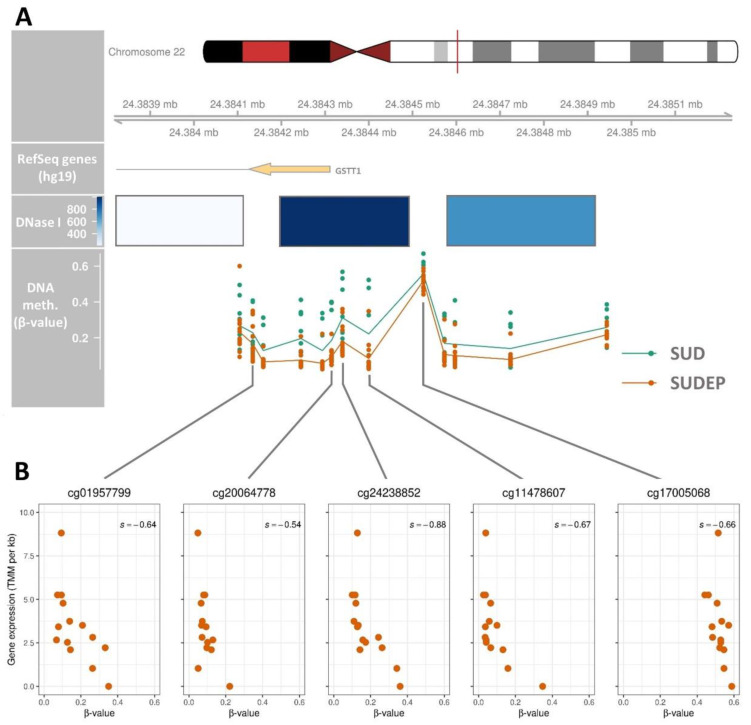
Regional differences of β-values from sudden unexplained death and sudden unexpected death in epilepsy cases overlapping the *GSTT1* region. (**A**) DNA methylation is shown for CpG positions in the differentially methylated region (DMR) overlapping the *GSTT1* region in ventricular tissues from sudden unexplained death (SUD) and sudden unexpected death in epilepsy (SUDEP). ENCODE DNase I sensitive regions are shown in blue with colour intensities corresponding to the signal strength. The lines connecting the points in the β-value track indicate the mean for each group. The DMR is located at chr22:24,384,105-24,384,944 covering 13 CpG positions. The colors in the chromosome structure correspond to chromosome staining bands and the vertical red line indicates the chromosomal location of the DMR. (**B**) The median of the three Trimmed Mean of M-values (TMM) per kb values from each individual was plotted against the β-value of each of the positions that were statistical significantly correlated (*p* ≤ 0.05) with *GSTT1* expression. Each CpG position is labelled with the CpG loci IDs according to the CpG nomenclature of Illumina.

One DMR between the SUD and SUDEP cases was identified using the recommended default kernel-smoothed false discovery rate (FDR) threshold of *DMRcate* (version 2.2.3) [38]. The DMR was located in proximity to the *GSTT1* gene (Figure 2A). To evaluate the regulatory relevance of the *GSTT1* DMR, DNase I sensitivity data from the Encyclopedia of DNA Elements (ENCODE) project was used for the mapping of likely *cis*-regulatory elements. Three clusters overlapped the *GSTT1* DMR with the second cluster scoring the maximal value for DNase I sensitivity. The expression data was also used to evaluate the regulatory relevance of the *GSTT1* DMR. The Spearman’s correlation coefficients among the methylation of each single CpG position within the DMRs and the median Trimmed Mean of M-values (TMM) per kb values were calculated for all CpG positions within the DMR. Among the 13 CpG positions overlapping the DMR in the *GSTT1* promoter region, five were negatively correlated (*p*-value ≤ 0.05) with the median TMM per kb for *GSTT1* (Figure 2B).

### 2.5. Differentially Methylated Regions between Sudden Unexplained Death and Sudden Unexpected Death in Epilepsy

Since only one DMR was detected, we decided to increase the FDR threshold to 0.001 well aware that it would increase the risk of type I errors. Six DMRs were identified between the SUD and SUDEP using the less stringent thresholds for DMR identification (Table 2). The Δβ among the CpG positions ranged from 0.10–0.19 within the six DMRs. Five of the DMRs overlapped a gene (*GSTT1*, *HLX*, *GSTM1*, *LOC391322*, or *TRIM5*) while the remaining DMR (chr22:24,348,549-24,348,715) was located in proximity to the *GSTT4* gene. Three of the DMRs (chr22:24,384,105-24,384,944, chr22:24,373,322-24,373,618, and chr22:24,348,549-24,348,715) were located within the same genomic region (<40 kb) as the genes, *GSTT4*, *LOC391322*, *GSTT1*, and the *GSTT1* antisense RNA 1 (*GSTT1-AS1*).

## 3. Discussion

SUD and SUDEP have similar circumstances of death and remain key diagnostic challenges in autopsies. Both of them are diagnoses of exclusion of all other possible causes of death, and the phenotypes of the deceased individuals are often heterogeneous and vague [39]. In this study, we aimed to assess and compare genetic and epigenetic patterns in cardiac tissues from SUD and SUDEP cases to highlight differences between the two groups.

The methylation patterns of consecutive CpG sites in SUD and SUDEP cases were assessed to identify DMRs. We show that a region upstream the *GSTT1* gene was differentially methylated between the SUD and SUDEP cases. By incorporating DNase I sensitivity and gene expression data, we found that the DMR is associated with *GSTT1* gene expression. The DNA methylation was negatively correlated with gene expression suggesting a repressive function of DNA methylation on *GSTT1* gene expression.

Only six DMRs were observed between the SUD and SUDEP cases. Five of these failed to pass the pre-determined FDR threshold. We performed no detailed analyses of these DMRs due to the relatively high Min. FDR for the regions (Table 2). However, two interesting patterns among the DMRs were observed. First, we observed that three DMRs were located in a genomic region from chr22:24,348,550-24,384,944 constituted by less than 40 kb. The region overlapped the glutathione transferases, *GSTT4, GSTT1*, and the *GSTT1* antisense RNA 1 (*GSTT1-AS1*), and thus it could be speculated that these DMRs are a part of the same biological feature. However, additional research is required to confirm this. Second, among the remaining three DMRs outside the 40 kb region, one of the DMRs was located in proximity to *GSTM1*, which encodes a protein that is part of the glutathione S-transferase (GST) superfamily just as *GSTT1* and *GSTT4*.

GSTs conjugate glutathione to toxic compounds such as products from oxidative metabolism, therapeutic drugs, and electrophilic molecules [40]. The normal function of GSTs is, therefore, essential for cellular detoxification and protection against oxidative stress. We cannot exclude that the observed differential methylation is a response to the intake of antiepileptic drugs in the SUDEP cases. However, a current study suggests an association between *GSTT1* dysregulation and dilated cardiomyopathy [41]. Heritable cardiomyopathies with a mild phenotype may cause SUD in victims where the autopsy findings are insufficient to determine a death cause [42]. Haas and colleagues reported decreased expression of *Gstt1* and *Gstt2* in a mouse model following transverse aortic constriction-induced heart failure and suggest that these genes are involved in adaptive or maladaptive cascades of the failing heart [41]. To our knowledge, there are no other direct associations between *GSTT1* and cardiac diseases associated with SUD. However, members of the GST superfamily have been associated with inhibition of the ryanodine receptor 2 [43]. The gene encoding this protein, *RYR2*, is one of the most commonly mutated genes in SUD [9,10], and variants within this gene are likely increasing the risk of SUDEP [30,44,45].

In addition to the association with cardiac disease, *GSTT1* null variants have been associated with epilepsy [46,47]. Metabolic dysregulation and oxidative stress is recognised as both a cause and consequence of epileptic seizures and may explain the relevance of *GSTT1* in SUDEP cases [48]. Based on the current knowledge about the GST superfamily, it cannot be excluded that the DMRs are associated with epilepsy and heart disease.

No statistically significant difference in allele frequencies was identified between the SUD and SUDEP cases. This may be explained by the heterogeneous nature of the diseases associated with SUD and SUDEP and the lack of statistical power due to the relatively small study population. Even though the aetiology of SUDEP is largely undetermined, the underlying pathophysiology is suspected to be multifactorial and complex [49,50,51,52]. SUD is likely caused by cardiac channelopathies such as LQTS, BrS, and CPVT, all of which are suspected to result from rare genetic variants [9,53]. It was, therefore, unlikely to identify a common causal genetic variant within the SUD and SUDEP cohorts. We did not identify genetic variation in proximity to the *GSTT1* gene that could explain the differential methylation between SUD and SUDEP cases. Neither did we find a correlation between genotypes in the *GSTT1* region and *GSTT1* RNA levels.

By using a larger study population, the power to detect significant differences in allele frequencies and differential methylation between the two groups would be higher. However, since both SUD and especially SUDEP are rare events, it is very difficult to obtain large study populations. In a 7-year period (2000–2006), only 50 definite and probable SUDEPs in the age group 1–35 years were identified among all residents in Denmark [16]. We observed that a part of the variation in methylation patterns was likely explained by age. This shows the importance of incorporating age as covariable in the statistical model for the identification of DMRs. We also investigated if the degree of post-mortem decomposition of the cardiac tissue could explain a part of the variation in methylation patterns. We did not find an association between the overall methylation patterns and the degree of post-mortem decomposition (data not shown). When using a relatively small sample size, it is difficult to obtain statistical significance after correction for multiple testing. Small methylation differences of single positions are therefore difficult to detect, but if the changes are consistent among a region, the statistical power will be greater [54]. Thus, we examined DMRs, which are arguably functionally more important compared to differentially methylated positions [55]. The use of FFPE tissue limits the quality of several molecular analyses due to the degradation of nucleic acids. However, array-based DNA methylation measurements of frozen and FFPE tissue show a good correlation [56,57].

We did not have the opportunity to compare our results with cardiac tissues from deceased individuals that died due to a non-cardiac event. Thus, we cannot determine if both SUD and SUDEP are differentially methylated compared to other deceased individuals.

With the use of a unique collection of cardiac tissue, it was demonstrated that the DNA methylation profiles of SUD and SUDEP were similar overall, except for the DMRs identified in proximity to genes encoding GSTs. Future studies are, therefore, needed to elucidate the role of DNA methylation in assumed cardiac deaths.

## 4. Materials and Methods

### 4.1. Study Population

The tissues were collected from autopsies of SUD and SUDEP cases that were carried out in 2009–2011 at the Section of Forensic Pathology, Department of Forensic Medicine, Faculty of Health and Medical Sciences, University of Copenhagen, Denmark. The cardiac tissues were fixed in 4% buffered formaldehyde for 48 hours and embedded in paraffin. Whole blood was stored at −20 °C in tubes coated with EDTA. Differences in clinical characteristics, such as age, sex, and circumstances of death, between SUD and SUDEP were assessed using the Fisher’s exact test for categorical variables and the Mann–Whitney U test for continuous variables.

### 4.2. Whole Genome Sequencing

WGS was performed as previously described [58]. Briefly, spin column-based DNA extraction from whole blood was performed, followed by WGS library preparation, and subsequent paired-end sequencing (2 × 150 bp) on a NextSeq 500 using the High Output kit v2 (Illumina, San Diego, CA, USA). Quality control of sequencing data and variant detection was performed using an in-house pipeline described in detail in [58].

Differences in allele frequencies between SUD and SUDEP cases were assessed with a two-sided Fisher’s exact test using the *fisher.test* function in the statistical software R (version 3.6.0) [59]. Statistically significant variants were defined as variants with a Bonferroni corrected *p*-value < 0.05.

### 4.3. Whole Transcriptome Sequencing

RNA was extracted from FFPE tissue of the anterior wall of the left cardiac ventricle. Two 20 µm FFPE tissue slides were used for each extraction. Excess paraffin was removed using xylene followed by a wash with 96–100% ethanol. Total RNA extraction was performed using the RNeasy FFPE Kit (Qiagen, Hilden, Germany) following manufacturer’s recommendations. Whole transcriptome library preparation was performed using the SMARTer^®^ Stranded Total RNA-Seq Kit v2-Pico Input Mammalian (Takara Bio Europe, St-Germain-en-Laye, France). Due to the fragmented nature of RNA extracted from FFPE tissue, no further fragmentation of the RNA was performed. cDNA synthesis was performed using random N6 primers followed by the addition of adapters and depletion of ribosomal cDNA. The quality and quantity of cDNA libraries were assessed using the Bioanalyzer 2100 instrument (Agilent Technologies, Santa Clara, CA, USA) and Qubit dsDNA HS Assay (Invitrogen, Waltham, MA, USA), respectively. Single-read sequencing (1 × 75 bp) on a NextSeq 500 using the High Output kit v2 (Illumina, San Diego, CA, USA) was performed. Biological triplicates were investigated for each of the 14 SUDEP cases. Whole transcriptome sequencing (WTS) data was only investigated in the SUDEP victims.

#### 4.3.1. Preprocessing and Alignment of RNA Data

The BCL files were converted to FASTQ files using *bcl2fastq* version 2.17.1.14 (Illumina, San Diego, CA, USA), and the sequencing quality was assessed using *FastQC* version 0.11.2 [60]. Consecutive stretches of low-quality bases (Q < 30) at the 5′ and 3′ termini as well as adapters were removed using *AdapterRemoval* version 2.1.3 [61]. Sequencing reads shorter than 20 bp were discarded.

The reads were aligned using *STAR* version 2.5.3a [62] using default settings except for two parameters. Reads aligned to multiple loci were classified as unmapped (--outFilterMultimapNmax 1), and the unannotated splice junction prediction was disabled (--alignIntronMax 1). The gene counts were obtained with *STAR* based on the human genome assembly GRCh37 (hg19) FASTA and GTF files from the GENCODE consortium release 27 [63].

#### 4.3.2. Normalisation of Read Counts

Reads in annotated non-coding, ribosomal, mitochondrial, or nuclear mitochondrial DNA segment (NUMT) sequences were excluded from analysis as previously described [64]. Normalisation of RNA data was performed according to the TMM method [65] using *EdgeR* [66] in R (version 3.6.0). Additionally, the read counts were normalised according to the gene length, and the normalised gene counts were presented as TMM per kilobase (kb).

### 4.4. Quantification of DNA Methylation

DNA from the FFPE tissues was extracted using five 20 µm slides from the tissue blocks using the QIAamp DNA FFPE Tissue Kit (Qiagen, Hilden, Germany) following the manufacturer’s recommendation with the only exception that the Proteinase K digestion step was conducted overnight until the tissue was completely dissolved.

The quality of the DNA extractions was evaluated using the Infinium FFPE QC Kit (Illumina, San Diego, CA, USA). Extracted DNA with an amplification cycle difference > 5 compared to the Infinium FFPE QC template DNA was excluded. The bisulfite conversion was performed to unveil methylated DNA bases using EZ DNA Methylation™ Kit (Zymo Research, Irvine, CA, USA) with 250–500 ng DNA. The elution of DNA was performed using 10 µL elution buffer. The eluate was subsequently restored using the Infinium HD FFPE DNA Restore Kit (Illumina, San Diego, CA, USA). The DNA methylation levels were measured using the Infinium MethylationEPIC BeadChip (Illumina, San Diego, CA, USA) following the manufacturer’s protocol (# 15,021,525 Rev. B). The slides were scanned using the iScan system (Illumina, San Diego, CA, USA).

All data analysis was conducted in R (version 3.6.0) using *tidyverse* (version 1.3.0) (Wickham et al., 2019), *patchwork* (version 1.1.1), and *RColorBrewer* (version 1.1-2).

The raw .idat files were imported into R using the *minfi* package (version 1.32.0) [67,68]. The samples with a mean detection *p*-value > 0.05 were excluded. The R package, *quantro* (v.1.20) [69], was used to perform a data-driven test to ensure that the assumption for conducting Quantile Normalisation of the data (no global differences in distributions between the SUD and SUDEP cases) was not violated. The signals were normalised using the *preprocessQuantile* function [68,70] and subsequently converted into methylation values. β- and M-values were calculated using the *getBeta* and *getM* functions, respectively. The three functions are all implemented in *minfi*. The PCA was conducted based on M-values and visualised using the *ggbiplot* package (version 0.55).

### 4.5. Differentially Methylated Regions

Regions of consecutive CpG positions with different methylation between SUD and SUDEP cases, i.e., DMRs, were identified using *DMRcate* (version 2.2.3) [38]. A CpG M-value matrix was annotated with chromosomal positions and test statistics using a design matrix with gender, age, and sample slide information as co-variables. The DMRs were identified using the recommended scaling factor = 2 and a minimum of three consecutive CpG positions in the DMR. The analysis of the DMRs was conducted using the *plyranges* (version 1.6.10) [38], *GenomicRanges* (version 1.38.0) [71], *org.Hs.eg.db* (version 3.10), *annotate* (version 1.64), and *TxDb.Hsapiens.UCSC.hg19.knownGene* (version 3.2.2) packages. Only regions with Δβ > 0.10 were considered as DMRs.

The *GSTT1* DMR was visualised using the *Gviz* package (version 1.30.3) [72] from R (version 3.6.0). The Hg19 RefSeq gene track was constructed using the *UcscTrack* function within the *Gviz* package and *org.Hs.eg.db*. Information of the DNase sensitive regions (wgEncodeRegDnaseClusteredV3) were downloaded from the University of California Santa Cruz (UCSC) Genome Browser. The regions were originally identified in 125 cell types and tissues as a part of the ENCODE project [73,74]. The Spearman’s correlation coefficients between the degrees of DNA methylation of the CpG positions and the gene expression in SUDEP were calculated using the *cor.test* function in R.

## Figures and Tables

**Table 1:** Descriptive data of the study population.

Characteristics	SUD (*n* = 9)	SUDEP (*n* = 14)	*p*-Value
Males, n (%)	6 (67)	10 (71)	1.000
Median age at time of death, years (range)	35 (0–50)	35 (3–49)	0.850
Witnessed death, n (%)	2 (22)	1 (7)	0.538
Performed toxicology screen, n (%)	6 (67)	11 (79)	0.643
No compounds detected, n (%)	2 (33)	2 (18)	0.584
One compound detected, n (%)	2 (33)	5 (46)	1.000
More than one compound detected, n (%)	2 (33)	4 (36)	1.000
Antiepileptic drug(s) detected, n (%)	0 (0)	8 (73)	0.009
Activity at time of death			
Sleep, n (%)	2 (22)	5 (36)	0.657
At rest, n (%)	3 (33)	3 (21)	0.643
Passenger in car, n (%)	1 (11)	0 (0)	0.391
Unknown, n (%)	3 (33)	6 (43)	1.000

SUD: Sudden unexplained death. SUDEP = Sudden unexpected death in epilepsy.

**Table 2:** Chromosomal regions with different methylation levels in sudden unexplained death compared to sudden unexpected death in epilepsy sorted by minimum false discovery rate.

Chr	Start	End	Size	CpG	Min. FDR	Δβ	Nearest Gene	Distance
chr22	24,384,105	24,384,944	840	13	3.81×10^–23^	0.15	*GSTT1*	0
chr1	221,053,841	221,054,825	985	10	6.04×10^–06^	−0.12	*HLX*	0
chr1	110,230,252	110,230,633	382	7	1.12×10^–05^	−0.14	*GSTM1*	0
chr22	24,373,322	24,373,618	297	3	1.17×10^–04^	0.19	*LOC391322*	0
chr11	5,959,658	5,959,945	288	3	4.61×10^–04^	0.11	*TRIM5*	0
chr22	24,348,549	24,348,715	167	3	6.15×10^–04^	−0.16	*GSTT4*	1290

Chr: Chromosome. Size: Size of the differentially methylated region (DMR) in bases. CpG: Number of CpG sites in the region. Min. FDR: Minimum false discovery rate (FDR) of the kernel-smoothed estimate of the DMR. Δβ: Mean β-value coefficient across the DMR between sudden unexplained death (SUD) and sudden unexpected death in epilepsy (SUDEP). Nearest gene: Nearest or overlapping gene for the DMR. Distance: Distance to nearest gene in bases. The DMR overlapped a gene region for values equal to zero.

## Data Availability

Data cannot be shared publicly because of data protection legislations (Danish Data Protection Agency no. 2011-54-1262). Data are available from the University of Copenhagen, Department of Forensic Medicine Institutional Data Access/Ethics Committee (contact via retsmedicinsk.institut@sund.ku.dk, University of Copenhagen, Department of Forensic Medicine) for researchers who meet the criteria for access to confidential data.
